# Cognitive Functions in Adolescent Girls with Anorexia Nervosa during Nutritional Rehabilitation

**DOI:** 10.3390/nu16203435

**Published:** 2024-10-10

**Authors:** Katarzyna Jowik-Krzemińska, Dagmara Dylewska, Aleksandra Pawlińska-Maćkowiak, Agnieszka Słopień, Marta Tyszkiewicz-Nwafor

**Affiliations:** 1 Department of Child and Adolescent Psychiatry, Poznan University of Medical Sciences, 27/33 Szpitalna St., 61-572 Poznan, Poland; asrs@gmail.com (A.S.); malamt@gmail.com (M.T.-N.); 2 Poznan University of Medical Sciences Doctoral School, 61-701 Poznan, Poland; 3 Department of Adult Psychiatry, Poznan University of Medical Sciences, 27/33 Szpitalna St., 61-572 Poznan, Poland; dagmaradylewska@gmail.com (D.D.); pawlinska.mackowiak@gmail.com (A.P.-M.)

**Keywords:** anorexia nervosa, cognition, eating disorders, adolescents

## Abstract

Background: The present study aimed to evaluate cognitive function and laboratory parameters in adolescent girls with anorexia nervosa (AN) before and after nutritional rehabilitation (NR) compared to healthy female peers (CG). Methods: We evaluated 36 girls with AN at two-time points, during acute malnutrition (AN1) and after NR, in a partially normalized weight status (AN2). We compared their cognitive functions and laboratory parameters to 48 healthy CG subjects. Cognitive function was assessed using a Cognitive Assessment Battery (CAB) assessment, depressive symptom levels were assessed using a Beck Depression Inventory (BDI) assessment, and eating disorders were assessed using an Eating Attitude Test (EAT-26). Results: The AN1 group scored better in total cognition, attention, estimation, and spatial perception than the CG group (*p* < 0.05), with scores increasing in the AN2 group. Shifting and visual perception values did not differ between the study groups (*p* = 0.677, *p* = 0.506, respectively). Laboratory tests showed no significant abnormalities and did not differ significantly between groups (*p* > 0.05). There was a negative correlation for EAT-26 and CAB in the AN1 group (rho = −0.43, *p* = 0.01), but not for BDI. Conclusions: Cognitive function in adolescent girls with AN was better than CG and correlated with EAT-26 score. These results highlight the high compensatory capacity of the adolescent body to maintain cognitive function despite severe malnutrition. Our results suggest that although normalization of body weight is crucial, other factors can significantly influence improvements in cognitive function. Cognitive deficits and laboratory tests may not be biomarkers of early forms of AN.

## 1. Introduction

Anorexia nervosa (AN) is a serious metabo-psychiatric condition characterized by disturbed body image, a strong fear of gaining weight, and dietary restrictions resulting in significant weight loss. Years ago, studies indicated a fairly stable incidence, but health professionals are seeing an increasing number of eating disorder (ED) patients receiving both outpatient and inpatient psychiatric care, especially during and after the COVID-19 pandemic [1,2,3,4,5]. The average age of onset of AN has steadily decreased over the past two decades: in the 1990s, it was 16–19 years, while in 2010, it was between 12 and 15 years. According to data from the UK, in children aged 10–14, the incidence of AN increased from 2.5 to 7.5 per 100,000 children. Similar trends have been observed in European countries, including Germany, Italy, and Portugal [6,7,8,9]. Among the reasons for this phenomenon is believed to be the growing influence of social media among young people, leading to increased comparison and the creation of unrealistic expectations about one’s body, and the influence of movements promoting anorexia-related behavior (so-called “pro-ana”) [10,11]. In parallel, there is a rise in social pressure to achieve success in the broadest sense and anxiety about the future [12], as well as an increase in morbidity and hospitalization due to AN during the geopolitical instability and lockdown situation of the COVID-19 period. The stress of armed conflict, social isolation, pandemic stress, health anxiety, and financial insecurity increase anxiety, which promotes the development of ED among adolescents [13,14,15].

The etiopathogenesis of AN is still unknown, and treatment results are unsatisfactory. It has been suggested that cognitive deficits may be a marker of the trait or endophenotype of the disorder, but reports on this subject are divergent [16,17,18]. Some researchers take the position that the axial symptoms of AN reflect neuropsychological dysfunction; for example, impaired assessment of body size has been described as a result of poor visuospatial abilities and cognitive and behavioral rigidity [19,20,21,22]. The above impairments can complicate the treatment process by hindering the implementation of new cognitive and behavioral skills. Previous literature highlights the unknown mechanism of cognitive deficits. It appears that they may be related to the somatic condition, as well as depressive symptoms [23]. Comorbid depression has attracted considerable research interest in the AN literature due to rates of comorbidity with this condition, ranging from 50 to 75 percent [24]. At the same time, cognitive impairment is a core feature of major depressive disorder. However, the scientific discourse lacks a clear answer to the question of the impact of depressive symptoms on the course of ED [25,26]. Furthermore, a poor somatic condition, manifesting in malnutrition, secondary hypothyroidism, and anemia, may also impair cognitive function [27,28]. Another vital reason for emphasizing cognitive functions in AN is the association between poorer neuropsychological functioning and poorer treatment outcomes [16,18,19,20,22]. Identifying such pathological, heritable, and condition-independent features is a potential key to developing highly effective therapeutic models based on an etiological pathway.

Nutrition plays a fundamental role in the treatment of AN, as it is essential for restoring average body weight, restoring micro- and macronutrient deficiencies, and improving the patient’s overall health [29]. Early initiation of nutritional rehabilitation (NR) leads to faster improvement in patients’ psychophysical state, which affects their ability to participate more fully in psychotherapy and other forms of treatment.

Therefore, the main objective of this study was to determine cognitive deficits in children with severe AN who required hospitalization in 24 h psychiatric wards. We hypothesized that children and adolescents with severe AN would have more profound cognitive deficits compared to their condition after NR and healthy peers. Including a group of recovered patients in the study allowed us to draw preliminary conclusions about whether the presumed cognitive impairment is an established trait after recovery from AN, one of the critical criteria that must be met for an agent to be considered an endophenotype. Based on clinical experience and intuitive understanding of AN, we assumed that patients should experience impairments in estimation, spatial perception, shifting, and all types of memory and show higher values in inhibition. In addition, we evaluated changes in serum laboratory profile (blood count, thyroid panel, vitamin D3 levels) at baseline and after NR. We hypothesized that after weight stabilization, the results of the parameters would improve significantly from the expected deviations from norms at the beginning of treatment. The evaluation also included correlations between cognitive test scores (CAB), changes in laboratory results, body weight, and depressive symptoms. We hypothesized that there would be a positive correlation between changes in cognitive domains and changes in body weight and a negative correlation with depression scales.

## 2. Methods

### 2.1. Participants

This prospective project was conducted between January 2021 and April 2024. Thirty-nine patients aged 10–18 years admitted to the child and adolescent psychiatry unit in the acute phase of AN participated in the study. All patients admitted to the hospital for AN were female during the study period. The study sample was anthropologically and demographically homogeneous. Written consent to participate in the study was obtained from all participants and their legal guardians.

AN diagnosis was made according to DSM-5 criteria after an interview with a child and adolescent psychiatrist. Exclusion criteria included physical illness and laboratory abnormalities unrelated to long-term food restriction. Between days 1 and 3 of admission, patients (AN1) underwent cognitive and anthropometric assessment (height and weight), psychological testing, and blood sampling. The same procedures were repeated at a second-time point, after completion of NR or partial normalization of body weight, on the day of discharge from the psychiatric ward (AN2).

Admitted patients were immediately placed on a NR program. Daily calorie intake was initially 2000 kcal and was gradually increased to 3000–3500 kcal, depending on the duration of hospitalization, laboratory results, and the rate of weight gain (approximately 1.0 kg per week was expected). Stabilization of health status was defined as follows:

patient consumption of 2000–3500 kcal per day;
exclusion of critical organ failure based on test results;
achievement of the discharge BMI determined at the start of hospitalization (according to centile grids appropriate for age, sex, and height);
confirmation by the attending physician of medical stabilization regarding somatic and psychological status, allowing further therapy in the outpatient setting.

During the project, eleven patients (28%) were discharged from the hospital at the request of a parent, against medical advice, before their psychosomatic state was fully restored. In this group, three patients were discharged on request within a few days of admission, so only the first examination (AN1) was performed on them without repeating it. Finally, thirty-six patients who underwent NR and spent at least 28 days in the hospital completed the study.

The control group (CG) was recruited at one of the local secondary schools. Forty-eight age- and sex-matched girls were included. Exclusion criteria in the CG were chronic somatic illness and psychiatric or neurodevelopmental disorders, including a history of ADHD or ED. Analogous to AN1, written consents were obtained from the participants and their legal guardians.

Weight and height were measured using a certified medical scale with a height gauge by a nurse to minimize the risk of measurement error. Body mass index (BMI) was calculated according to the formula BMI = weight (in kg)/height^2^ (in m^2^) [30]. The percentage of ideal body weight (%IBW) was calculated according to the Lorentz formula (for women: (height − 100) − ((height − 150)/2)) [31].

The Bioethics Committee approved study protocol 476/20 of the University of Poznan Medical School (17 June 2020). All procedures were carried out following the 1964 Declaration of Helsinki.

### 2.2. Biochemical Analysis

Venous blood was collected in the morning from fasting AN1 patients (8 h after the last meal) and analyzed in the hospital laboratory. An analogous collection was performed after NR, 1–3 days before planned discharge. Blood serum collected from CG was analyzed in the same hospital laboratory. Blood counts (red blood cells (RBCs), hemoglobin (HGB) and hematocrit (HCT), white blood cells (WBCs) and platelets (PLTs)), glucose and insulin levels, thyrotropic hormone (TSH), free thyroxine (ft4), and vitamin D (1.25(OH)2D3) levels were examined. From the glucose and insulin results, the homeostatic insulin resistance assessment model index (HOMA-IR) was calculated: insulin (mU/mL) × glucose (mg/dL).

### 2.3. Cognitive Assessment

Cognitive functions were tested using the CogniFit Cognitive Assessment Battery (CAB), a set of computer-assessed neuropsychological tests commonly used in research protocols. According to the manufacturer, the FDA registration number is 3017544020*, and the EUDAMED (European Database on Medical Devices) UDI-DI registration is 00860009958104 [32]. The CAB includes experiments validated against various standardized neuropsychological tests, including the full automatic Cambridge Neuropsychological Test battery, Raven’s Standard Progressive Matrices, Wisconsin Card Sorting Test, Continuous Performance Test, and STROOP Test. Tests of its reliability have been demonstrated in previous studies, yielding corresponding measures of good internal consistency (Cronbach’s alpha = 0.85–0.88), and test–retest reliability (r = 0.69–0.92). The assessment uses clinical scales and tests validated for the age and gender of the patient or participant. The set of tasks is designed to assess 22 basic cognitive abilities divided into five main domains: CA—Perception (Auditory Perception, Estimation, Recognition, Spatial Perception, Visual Perception, Visual Scanning); CA—Attention (Divided Attention, Focus Attention, Inhibition, Updating); CA—Reasoning (Planning, Processing Speed, Shifting); CA—Memory (Phonological Short-Term Memory, Contextual Memory, Short-Term Memory, Non-Verbal Memory, Visual Short-Term Memory, Working Memory, Naming); CA—Coordination (Hand–Eye Coordination, Response Time).

This neuropsychological test takes 30–40 min and is conducted online. All participants took the test on a laptop in a separate room, using in-ear headphones to ensure comfort and maximum reduction in external distractions.

### 2.4. Psychometric Tests

All study participants completed anonymous tests—the Beck Depression Inventory (BDI), assessing the severity of depressive symptoms, and the Eating Attitudes Test (EAT-26), assessing eating attitudes [33,34]. Both questionnaires were validated in the Polish version, obtaining Cronbach’s α indices of 0.90–0.92 and 0.90, respectively. In both questionnaires, the higher the score, the greater the severity of depressive symptoms or eating disorders, respectively, with presumed cut-off points of 11 in the BDI and 20 in the EAT-26. Patients with AN were assessed twice at inclusion and the end of the program, while the CG group was evaluated once.

### 2.5. Statistics

Analyses were conducted using statistical software R, version 4.1.2. The normality of distributions was checked with a Shapiro–Wilk’s test, as well as with the values of skewness and kurtosis. Variance homogeneity was assessed using a Levene’s test. Independent comparisons were conducted with a Student’s *t*-test for independent groups, Welch’s *t*-test for independent groups, Mann–Whitney’s U test, or Pearson’s Chi-square test, as appropriate. Paired comparisons were conducted with paired *t*-test or Wilcoxon test, as appropriate. Correction of *p* values due to multiple comparisons was implemented using Benjamini and Hochberg adjustment for each type of comparison. The effect size was calculated for each comparison with either Cohen’s D or r for the Wilcoxon test (Z statistic divided by the square root of sample size), with 95% confidence intervals. Either an independent or paired version was applied, as appropriate. Spearman’s rho coefficient was used for correlation analyses due to non-normal distributions of some variables. Linear regression models were fitted to understand the impact of selected predictors on CAB. After the initial selection of parameters for multivariate models, a stepwise procedure was employed for the final variables selection. All statistical calculations assumed alpha = 0.05. The tests comparing EAT-26, CAB, and BDI values between groups achieved test power, respectively: 100%, 59%, and 99.6%.

## 3. Results

### 3.1. Anthropometric Data

Table 1 compares demographic and clinical data between AN1, AN2, and CG. The mean age of onset of AN in patients was 14.5 years, while the mean age of CG was 15.5 years. Patients with acute malnutrition (AN1) had lower BMI, body weight, and %IBW than patients with partial normalization of body weight (AN2). At the same time, AN2 patients had lower scores in these parameters than the CG.

The percentage of patients with menarche and a history of menstrual bleeding in the AN1 group was 69.4%, while the corresponding percentage among CG was 100.0%. All patients in the AN1 group did not menstruate during hospitalization and for at least three months before hospitalization, while the rate of menstruating patients in the CG group was 100.0%.

The average length of hospitalization of patients with AN was 68 days. Analysis of hospital records within two years of the first hospitalization (at the time of the aforementioned study) showed that 15 (38.5%) were readmitted to the ward due to exacerbation/recurrence of AN symptoms.

### 3.2. Cognitive–Psychological Functioning

**Figure 1:**
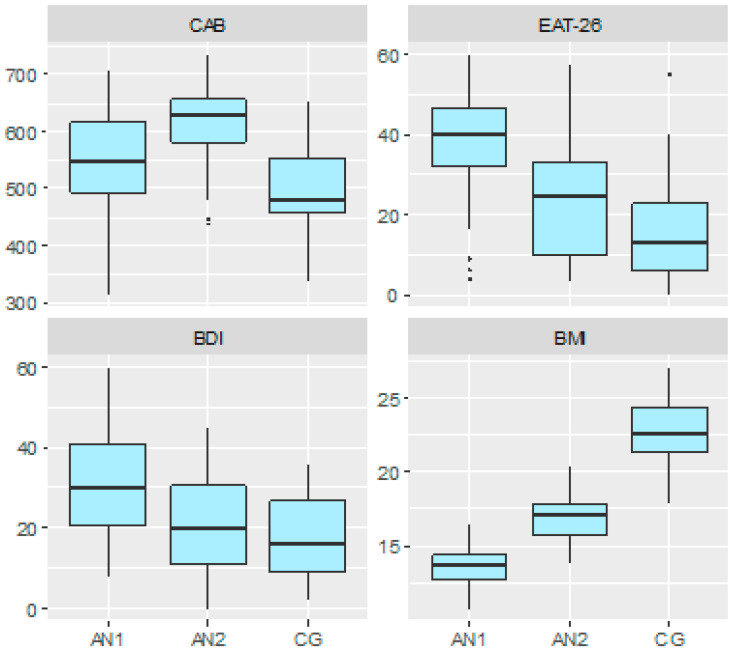
Boxplots presenting the distribution of selected parameters among patients with low body weight (AN1), after nutritional rehabilitation (AN2) and among the control group (CG).

Despite the malnutrition, the AN1 group had statistically significantly higher total scores in the CAB than the CG. Moreover, the scores increased with the normalization of body weight (AN2). The same statistically significant tendency was observed in the studied groups concerning CA—Perception and CA—Attention. In CA—Reasoning, the AN1 group had lower scores than the CG and normalized it with body weight gain (*p* = 0.036). In contrast, CA—Memory did not differ between AN1 and CG but improved statistically significantly with weight normalization and was higher in the AN2 group than in CG. The same tendency was observed for CA—Coordination. (Table 2). A significant difference in BDI values was observed between AN1 and CG (*p* < 0.001). At the same time, there was a significant decrease throughout treatment (*p* < 0.001). The outcome of the AN2 and CG groups did not differ statistically significantly. Regarding EAT-26, differences were statistically significant, with the highest scores in AN1 and the lowest in CG (Figure 1).

The detailed analysis of single cognitive functions presented in Table 3 showed that despite the malnutrition, AN1 scored significantly higher than CG in estimation, recognition, spatial perception, focus attention, planning, visual short-term memory, contextual memory, and short-term memory. During NR (AN1 vs. AN2), auditory perception, recognition, visual perception, visual scanning, focus attention, updating, processing speed, phonological short-term memory, naming, contextual memory, short-term memory, working memory, response time, and hand–eye coordination improved statistically significantly. Estimation, spatial perception, divided attention, inhibition, planning, visual short-term memory, and non-verbal memory did not change with increasing body weight and remained higher as in the health peers (AN2 vs. CG). The shift value did not change between the AN1 and AN2 groups and remained without statistical difference compared to CG (*p* = 0.634).

### 3.3. Biochemical Blood Results

In laboratory tests (Table 4), the mean level of 1.25(0H)2D in each group was below reference values but significantly higher in the AN1 than AN2 group. At the same time, the AN1 group was not statistically different from the CG (*p* = 0.066).

RBCs, HGB, and HCT in all three groups were within reference values. They did not change during weight rehabilitation (AN1 vs. AN2), and the results remained significantly lower than in CG after weight normalization.

Fasting glucose and insulin concentrations and HOMA-IR scores were significantly lower among patients in AN1 than in AN2. No statistically significant differences were observed between AN2 and CG. TSH values were not significantly different among all three groups. However, FT4 values were lower among patients in AN1 and AN2 than in CG with high strength of change as measured by the r effect size for the Wilcoxon test (AN1 vs. CG r = 0.57 CI 95 [0.39; 0.73] and AN2 vs. CG r = 0.75 Cl 95 [0.61; 0.83], respectively). 

### 3.4. Correlations between Cognitive Function Scores and Psychometric Variables

Correlations between CAB/CA parameters and BDI/EAT-26 were verified in patients with low body weight (AN1) and after partial normalization of body weight (AN2) and CG. A negative correlation was noted for EAT-26 and CAB in the AN1 group (rho = −0.43, *p* = 0.01) (Table 5).

### 3.5. Linear Regression

In univariate models of AN1, EAT-26, higher by 1, translated into CAB, lower by 2.77, β = −2.77 CI95 [−4.85; −0.69], *p* = 0.011 (Supplementary Materials—Table S1). The initial selection of variables for multivariate models was based on the magnitude of effect size for the difference between AN1 and AN2, *p*-value from univariate models, and clinical knowledge on potential predictors of CAB. Multivariate models in version 1 assumed the following variables: age, BMI, %IBW, glucose, HOMA-IR, EAT-26, and BDI. After utilizing the stepwise selection procedure, the final multivariate models comprised age, BMI, and EAT-26. EAT-26 impacted CAB statistically significantly in AN1. Its value, higher than 1, translated into CAB, lower by 2.34, β = −2.34 CI95 [−4.47; −0.21], *p* = 0.032. The impact of EAT-26 in AN2 on CAB was measured with a significance level of *p* = 0.053 (β = −1.49 CI95 [−3.00; 0.02], *p* = 0.053). Multivariate models in version 2 assumed the following variables: age, body weight, %IBW, glucose, HOMA-IR, EAT-26, and BDI. After utilizing the stepwise selection procedure, the final multivariate models consisted of body weight, glucose, and EAT-26. Body weight, higher by 1 kg, translated into CAB, lower by 5.83, β = −5.83 CI95 [−11.63; −0.03], *p* = 0.049 in AN1. In AN2, the impact of body weight and EAT-26 on CAB were measured with significance levels of *p* = 0.087 and *p* = 0.096, respectively (β = −2.85 CI95 [−6.14; 0.44], *p* = 0.087 and β = −1.27 CI95 [−2.77; 0.24], *p* = 0.096, respectively), Table 2. The model’s fit in version 1 in a group with low body weight was assessed with R2 and adjusted R2, with the outcomes of 24.5% and 17.5%, respectively. The model’s fit in version 2 in a group with low body weight assessed with R2 and adjusted R2 was 27.4% and 20.6%, respectively. Multicollinearity was assessed with VIF indicators, which was below 2.00 for all predictors and all multivariate models.

## 4. Discussion

The study presented here is the first part of a larger analysis of the psychophysical status of pediatric patients with AN and the dynamics of change during NR. Our initial hypothesis was that patients with severe AN would have cognitive deficits compared to patients after NR and the healthy population. We reasoned that reduced caloric and nutrient intake was crucial for the regression of cognitive functioning. Meanwhile, contrary to these expectations, the total mean CAB score and most studied cognitive parameters were higher in patients in the acute phase of AN and after NR than in healthy adolescents. At the same time, patients had significantly better cognitive performance after NR (AN2) than initially (AN1). However, some parameters, such as estimation, inhibition, planning, and spatial perception, did not change with weight gain, although they were higher than in the CG. Of the 22 studied cognitive functions, only shifting did not differ between groups. This is puzzling since the literature has divergently described the effects of ED on academic achievement and performance—both negatively [35,36] and positively [37]. However, this would be consistent with the fact that EDs are reported to be more common in individuals with high intelligence, perfectionism, or higher school achievement [38,39,40,41].

To our knowledge, short-term memory parameters have not yet been compared in adolescents with AN undergoing nutritional treatment. Our subjects had significantly better parameters despite the disease than CG, and these parameters still improved after NR. Meanwhile, Terhoeven et al. and Nikendei et al., in independent studies, argued that adult patients with AN showed worse results of this parameter than a healthy control group, and the deviations persisted despite restoration of normal body weight [42,43]. Our group differed significantly from those mentioned by age (<18 years old) and the fact of longitudinal follow-up, which allowed us to limit the influence of personal variables on the results after NR.

Inhibition, understood as the ability to control impulsive reactions, contributes to planning, goal-setting, and inhibition of automatic responses to stimuli and develops in early to middle adolescence. It has been hypothesized that the degree of proactive inhibition is related to how well an individual tolerates uncertainty and dominates reactive inhibition in patients with AN [44,45,46]. Shen et al. proved significant differences between intentional and reactive inhibition during adolescence, which integrate with age [47]. Both Suttkus’ and Tenconi’s work showed no differences between patients with AN and CG, but the studies included adolescents and young adults (mean age 19 in Tenconi’s work, 26 in Suttkus’ article) with a correspondingly long duration of illness (mean 24 months in Tenconi’s) [48,49]. We show that adolescents in the acute phase of AN had similar inhibition scores as after NR, while not significantly different from healthy peers, which would confirm some independence of clinical and nutritional status. However, longitudinal evaluations are lacking in the literature, so we can only speculate that inhibition worsens with disease progression and duration.

Surprising results were obtained for the parameters of estimation and spatial perception, which, contrary to the intuitive assumption that they would be impaired due to the primary symptoms of ED (impaired perception of the shape and size of one’s own body), are significantly better than in healthy subjects. Contrary to our initial assumptions, this is a significant difference, as earlier studies by Scarpin and Guardia confirmed impaired imaginal processes and spatial reference in AN compared to healthy subjects [50,51]. We do not know whether this mechanism is primary or secondary to the symptoms and consequences of AN, especially since the strength of the difference between AN and CG for estimation was moderate, and the values did not change during NR. This prompts us to extend further research on the mechanisms of impaired body representation in patients with high global spatial perception and to estimate and study the relationship and influence of factors other than body weight on the above variables.

AN is associated with poor cognitive flexibility, referring to the ability to change thoughts or actions according to situational demands. These findings are mostly observed in adults, and conclusions, including those of adolescents, are less consistent [20,52,53,54,55]. Our analysis showed that shifting did not change before and after treatment. We believe that adolescence, with its intense development of the central nervous system and cognitive abilities, influences the development of executive functions and protects against the neurodegenerative processes observed in adults with AN. This would be consistent with the lack of differences in working memory between AN1 and CG. Samanta Brooks et al. hypothesize that people with AN have an excessive and thus dysfunctional working memory, similar to other studies on the neural correlates of AN [56,57,58]. Some, however, indicate deficits [22,59] or no differences [42,60] between people with AN and overeaters or healthy individuals.

We examined a baseline biochemical panel from blood to better explore the relationship between cognitive functioning and its component parameters. Most clinicians, both pediatricians and psychiatrists, expect significant abnormalities in laboratory tests in patients with AN, especially as children have a lower percentage of body fat than adults, and the consequences of weight loss may be more severe because of this. In the course of AN, the typical changes described in the blood picture are anemia, leukopenia, and, less frequently, thrombocytopenia. Cell lineage changes are due to chronic malnutrition and bone marrow hypometabolism, myelosuppression, or reduced erythropoietin (EPO) or thrombopoietin (TPO) secretion [61,62]. Meanwhile, our patients, despite the need for hospital treatment due to their severe medical condition and severe malnutrition, did not show significant deviations in their blood biochemistry results. Both blood morphology parameters, such as red blood cell (RBC, HGB, HCT), WBC, and PLT lineage indices, did not exceed reference standards, indicating anemia, reduced cellular immunity or risk of pancytopenia. As shown in previous literature, the frequency and severity of hematological abnormalities associated with bone marrow hypoplasia may be related to disease duration and severity of malnutrition [51].

Similarly, despite extreme malnutrition, neither in the initial hospitalization nor subsequently did glucose and insulin levels deviate from laboratory norms and excluded morning hypoglycemia or insulin resistance (HOMA-IR < 2), which differs from previous literature reports of frequent hypoglycemia in patients [63].

Similarly, despite extreme malnutrition, neither in the initial hospitalization nor subsequently did glucose and insulin levels deviate from laboratory norms and excluded morning hypoglycemia or insulin resistance (HOMA-IR < 2), which differs from previous literature reports of frequent hypoglycemia in patients [64,65,66]. These significant differences between our study and prior literature may be due to the young age of the patients, the relatively short duration of the disease (first ED episode), and the high compensatory capacity of the adolescent body to maintain homeostasis despite a persistent negative energy balance. Moreover, this represents a very favorable prognostic factor, as hypoglycemic incidents and reduced liver volume are predictors of a severe and complicated course of the disease. Thyroid function tests in patients showed that both TSH and FT4 were normal in the AN1 and AN2 groups and CG. This is consistent with previous literature, which provides evidence that the pathology of the hypothalamic–pituitary–thyroid axis is not associated with menstrual retention [67].

In addition, our analysis assessed serum vitamin D3 levels. Although both AN and CG were deficient at the beginning of the study, the levels were nevertheless higher in female patients than in the healthy population. At the same time, the levels decreased during hospitalization and NR. We partly relate this to the lack of supplementation in the treatment protocol used and the conditions of stay (closed ward, limited outdoor and sun exposure). However, this is only a partial explanation for the above phenomenon: a review of the literature showed that, in most cases, a vitamin D deficit was observed in patients with AN compared to CG, even if their intake was the same, which was explained by reduced adipose tissue, reduced metabolic clearance and reduced vitamin D3 uptake by adipose tissue. This is important not only because of its association with the skeletal status of adolescent patients but also with the occurrence of depression and other psychiatric disorders [68,69,70,71]. We suggest that the relatively high levels, higher than in the healthy population, may be due to the claimed high attention to health and adequate vitamin and mineral supplementation in people with AN.

Malnutrition, secondary to AN, is a chronic condition in which metabolic and functional adaptive mechanisms develop to allow survival despite very low body weight and organ complications. We believe that in the course of AN in children and adolescents, despite low BMI, abnormalities in biochemical tests may not be clinically significant, reflect the severity of the disease, and may not be a reliable predictor of emergency conditions in the course of AN, such as cardiac arrhythmias or acute pancreatitis. This indicates a high adaptive capacity of the young organism.

We initially hypothesized that cognitive function would improve with normalization of body weight and restoring adequate nutrition and hydration, but our analyses did not yield the predicted conclusive result. As in the Hemmingsen et al. project, we did not confirm a positive correlation between BMI and improved cognitive function during NR. In our analysis, CAB level correlated negatively with body weight in the acute phase of AN [72]. At the same time, we did not show an association between depressive symptoms and CAB, although the severity of eating-disorder symptoms already correlated significantly with cognitive level. It can be speculated that other variables not included in the study have a significant impact on the improvement of cognitive performance after weight normalization. This could be, for example, the psychological well-being of the subject, which, due to illness and false beliefs or thought disorders, decreases with increasing body weight, as well as environmental factors or other pathophysiological variables. We link the refutation of the hypothesis of cognitive improvement depending on nutrition as measured by body weight and BMI to the clinical characteristics of individuals with AN, who are characterized by high levels of perfectionism, control, and obsessive–compulsive symptoms, which are manifested in the adolescent’s daily life by regular and systematic study, high academic performance and sporting and artistic achievements. This is supported by a cohort study in which high school achievement and higher parental education were associated with increased ED risk [73].

The severity of depressive symptoms was significantly higher in AN1 patients than in CG and improved during AN treatment, reaching values similar to healthy peers. At the same time, we did not detect significant correlations between them and cognitive functions, despite the intuitive assumption that cognitive parameters would improve with a reduction in depressive symptoms. Interestingly, they improved with the withdrawal of eating disorder symptoms, once again indicating the enormous complexity of AN processes and mechanisms.

The main limitation of the presented study is the relatively small number of participants during the follow-up period, which may weaken the study’s strength. However, the studies discussed above usually contain a similar group of subjects. Moreover, our study includes a very homogeneous group of patients regarding age, menstruation, number of episodes, and treatment method, which may improve its quality. Importantly, it is essential to remember that AN is a multifactorial disorder with a complex metabolic-psychiatric-social etiopathogenesis. In the present study, we focused on examining specific factors due to the breadth of the topic, omitting the influence of perfectionism level, anxiety, obsessive–compulsive symptoms, attachment style, or family functioning.

In the presented study, we did not include an analysis of sex hormone levels, although some studies described the link between hormonal and cognitive functioning in female patients with AN. Moreover, the CG was slightly older than AN by an average of 1 year, which could also be a potential factor for differences in maturation level and cognitive parameters. However, we believe the age difference was insignificant given the higher scores in the younger, sicker girls.

## 5. Conclusions

The main finding of this study is that the initially assumed cognitive impairment in adolescents with AN compared to healthy peers did not occur at all, and parameters continued to improve after NR treatment. These results suggest that some variables may be related to the patient’s nutritional status. However, these are more complex correlations, and cognitive functioning must depend on many other factors not considered in previous studies. Adolescent patients with AN tend to have a shorter duration of illness, a shorter duration of hunger, and a higher rate of treatment-seeking (parental treatment seeking). The teenagers we studied required hospitalization for AN for the first time, indicating that this was their first acute episode of the disease. This probably explains the differences between our study and the literature. Years of malnutrition alter brain structure and function and cause permanent cognitive deficits analogous to scarring of brain structure. At the same time, morphological changes in brain structure take a different course, and the reduction in gray matter volume is more significant in adolescents than in adults. To date, there is no clear consensus in the literature about the effect of AN on neuropsychological function. Most studies have focused on adult patients with a long history of the disorder, leaving a gap in understanding adolescent patients with a shorter duration of AN and their experiences of starvation. Therefore, therapeutic interventions should be tailored to the duration of illness, degree of chronification, and associated neurocognitive deficits. The above study shows that cognitive deficits and laboratory tests may not be biomarkers of early-stage AN.

The results suggest that weight gain does not affect cognitive function. Investigating the dynamics of cognitive changes and the factors affecting them may be an essential target for future research. Neuropsychological functioning in the disease may be an important measure of prognosis, and a complete understanding of the cognitive function of patients with AN may provide more effective screening of disease risk groups, creating more tailored prevention and treatment programs.

## Figures and Tables

**Table 1:** Demographic characteristics of patients with anorexia nervosa. Anthropometric parameters in low body weight (AN1), after nutritional rehabilitation (AN2), and in the control group (CG).

Variable	Group M ± SD/Me (Q1; Q3)			AN1 vs. AN2		AN1 vs. CG		AN2 vs. CG	
	AN1 (n = 36)	AN2 (n = 36)	CG (n = 48)	Cohen’s D/Wilcoxon Effect Size r (95% CI) *	Adj *p*	Cohen’s D/Wilcoxon Effect Size r (95% CI) **	Adj *p*	Cohen’s D/Wilcoxon Effect Size r (95% CI) **	Adj *p*
Age	14.53 ± 1.59	-	15.52 ± 0.97	-	-	−0.78 (−1.23; −0.33)	0.005 ^2^	-	-
Height [m]	1.61 (1.56; 1.65)	-	1.65 (1.62; 1.69)	-	-	0.34 (0.15; 0.53)	0.0065	-	-
BMI [kg/m^2^]	13.51 ± 1.40	16.89 ± 1.51	22.70 ± 2.31	−2.27 (−2.94; −1.59)	<0.001 ^3^	−4.66 (−5.49; −3.83)	<0.001 ^2^	−2.90 (−3.51; −2.28)	<0.001 ^2^
Body weight [kg]	34.46 ± 5.51	43.16 ± 7.02	62.27 ± 6.61	−1.29 (−1.58; −0.99)	<0.001 ^3^	−4.51 (−5.32; −3.69)	<0.001 ^1^	−2.81 (−3.42; −2.20)	<0.001 ^1^
IBW	55.50 (53.19; 57.62)	-	57.25 (56.00; 59.62)	-	-	0.34 (0.14; 0.54)	0.005	-	-
%IBW	0.63 ± 0.07	0.79 ± 0.08	1.08 ± 0.11	−2.04 (−2.62; −1.47)	<0.001 ^3^	−4.82 (−5.67; −3.96)	<0.001 ^2^	−3.01 (−3.64; −2.37)	<0.001 ^1^

Data presented as M ± SD or Me (Q1; Q3), M ± SD was used when distribution in each group was normal, and difference between AN1 and AN2 had normal distribution, Me (Q1; Q3) was used otherwise. M—mean, SD—standard deviation, Me—median, Q1—first quartile, Q3—third quartile, CI—confidence interval. Comparisons were made with Student’s *t*-test for independent samples ^1^, Welch *t*-test for independent samples ^2^, Mann–Whitney U test, paired *t*-test ^3^. *p* values were corrected with Benjamini and Hochberg adjustment in each comparison type; this Table presents corrected *p* values (adj *p*). * Variants for paired tests.; ** Variants for independent tests.

**Table 2:** Values of depression indices (Beck Depression Inventory, BDI), eating disorders (Eating Attitude Teat, EAT-26), and cognitive function parameters measured with the Cognitive Assessment Battery (CAB) in adolescents with anorexia nervosa at low body weight (AN1) and nutritional rehabilitation (AN2) and in a healthy control group (CG).

Variable	Group M ± SD/Me (Q1; Q3)			AN1 vs. AN2		AN1 vs. CG		AN2 vs. CG	
	AN1 (n = 36)	AN2 (n = 36)	CG (n = 48)	Cohen’s D/Wilcoxon Effect Size r (95% CI) *	Adj *p*	Cohen’s D/Wilcoxon Effect Size r (95% CI) **	Adj *p*	Cohen’s D/Wilcoxon Effect Size r (95% CI) **	Adj *p*
CAB	550.34 (492.76; 616.50)	629.57 (582.94; 656.24)	483.02 (457.70; 555.48)	0.85 (0.77; 0.87) ^2^	<0.001 ^1^	0.27 (0.04; 0.48) ^2^	0.037	0.59 (0.42; 0.72) ^2^	<0.001
CA—Perception	634.00 (563.00; 705.75)	681.50 (640.00; 733.75)	488.33 (458.00; 543.50)	0.55 (0.26; 0.77) ^2^	0.002 ^1^	0.54 (0.33; 0.70) ^2^	<0.001	0.70 (0.56; 0.81) ^2^	<0.001
CA—Attention	612.50 (547.50; 650.00)	660.00 (602.25; 690.25)	531.00 (527.47; 605.50)	0.59 (0.32; 0.80) ^2^	0.001 ^1^	0.27 (0.06; 0.47) ^2^	0.036	0.50 (0.29; 0.66) ^2^	<0.001
CA—Reasoning	599.00 (556.25; 646.25)	654.00 (620.00; 686.75)	622.00 (620.60; 674.50)	0.73 (0.56; 0.85) ^2^	<0.001 ^1^	0.27 (0.05; 0.49) ^2^	0.036	0.12 (0.01; 0.34) ^2^	0.348
CA—Memory	512.50 (435.00; 587.50)	619.00 (568.75; 647.25)	460.60 (455.80; 592.00)	0.83 (0.73; 0.87) ^2^	<0.001 ^1^	0.10 (0.01; 0.34) ^2^	0.468	0.46 (0.27; 0.64) ^2^	<0.001
CA—Coordination	391.00 (307.00; 500.00)	528.00 (424.25; 608.50)	315.33 (307.00; 400.50)	0.78 (0.63; 0.87) ^2^	<0.001 ^1^	0.21 (0.02; 0.41) ^2^	0.091	0.51 (0.32; 0.68) ^2^	<0.001
EAT-26	40.00 (31.75; 46.25)	24.50 (10.00; 33.00)	13.00 (6.00; 22.50)	0.76 (0.57; 0.86)	<0.001 ^1^	0.63 (0.46; 0.75)	<0.001	0.26 (0.04; 0.47)	0.033
BDI	30.00 (20.50; 41.00)	20.00 (11.00; 30.50)	16.00 (9.00; 26.50)	0.69 (0.47; 0.86)	<0.001 ^1^	0.44 (0.26; 0.61) ^2^	<0.001	0.12 (0.01; 0.34) ^2^	0.351

Data presented as M ± SD or Me (Q1; Q3), M ± SD was used when distribution in each group was normal, and the difference between AN1 and AN2 had normal distribution; Me (Q1; Q3) was used otherwise. M—mean, SD—standard deviation, Me—median, Q1—first quartile, Q3—third quartile, CI—confidence interval. Comparisons were made with Wilcoxon test ^1^. *p* values were corrected with Benjamini and Hochberg adjustment in each comparison type; this Table presents corrected *p* values (adj *p*). In the case of Mann–Whitney U test/Wilcoxon, the r effect size for Wilcoxon test ^2^ was given. * Variants for paired tests. ** Variants for independent tests.

**Table 3:** Values of single cognitive functions measured with the Cognitive Assessment Battery (CAB) in adolescents with anorexia nervosa at low body weight (AN1), after nutritional rehabilitation (AN2) and in a healthy control group (CG).

Main Variable	Variable	Group M ± SD/Me (Q1; Q3)			AN1 vs. AN2		AN1 vs. CG		AN2 vs. CG	
		AN1 (n = 36)	AN2 (n = 36)	CG (n = 48)	Cohen’s D/Wilcoxon Effect Size r (95% CI) *	Adj *p*	Cohen’s D/Wilcoxon Effect Size r (95% CI) **	Adj *p*	Cohen’s D/Wilcoxon Effect Size r (95% CI) **	Adj *p*
CA—Perception	Auditory Perception	567.50 (447.25; 670.50)	688.50 (622.00; 742.25)	526.27 (523.63; 662.00)	0.73 (0.56; 0.84) ^6^	<0.001 ^4^	0.01 (0.00; 0.27) ^6^	0.917	0.43 (0.22; 0.62) ^6^	<0.001
	Estimation	619.50 (379.75; 696.75)	676.00 (505.50; 703.50)	514.87 (436.50; 573.50)	0.16 (0.01; 0.45) ^6^	0.375 ^4^	0.26 (0.04; 0.48) ^6^	0.037	0.44 (0.21; 0.64) ^6^	<0.001
	Recognition	561.00 (408.50; 634.75)	633.00 (577.50; 706.00)	387.87 (360.00; 545.00)	0.70 (0.49; 0.85) ^6^	<0.001 ^4^	0.27 (0.04; 0.48) ^6^	0.036	0.57 (0.39; 0.72) ^6^	<0.001
	Spatial Perception	613.00 (415.25; 647.25)	611.00 (308.00; 707.25)	258.47 (202.50; 317.00)	0.21 (0.01; 0.54) ^6^	0.240 ^4^	0.55 (0.36; 0.71) ^6^	<0.001	0.50 (0.30; 0.67) ^6^	<0.001
	Visual Perception	657.50 (589.25; 732.25)	694.50 (644.50; 737.25)	578.00 (577.13; 745.00)	0.47 (0.20; 0.68) ^6^	0.007 ^4^	0.09 (0.00; 0.32) ^6^	0.506	0.26 (0.05; 0.48) ^6^	0.034
	Visual Scanning	505.00 (263.50; 628.50)	599.50 (434.75; 631.00)	532.00 (496.50; 610.00)	0.52 (0.26; 0.75) ^6^	0.003 ^4^	0.18 (0.01; 0.41) ^6^	0.156	0.07 (0.00; 0.29) ^6^	0.634
CA—Attention	Divided Attention	787.50 (781.75; 800.00)	795.00 (787.50; 800.00)	785.87 (779.50; 795.50)	0.32 (0.03; 0.60) ^6^	0.081 ^4^	0.11 (0.01; 0.32) ^6^	0.456	0.27 (0.05; 0.49) ^6^	0.027
	Focus Attention	604.00 (436.00; 650.25)	629.00 (587.50; 680.25)	431.93 (399.00; 582.50)	0.63 (0.38; 0.81) ^6^	<0.001 ^4^	0.26 (0.05; 0.47) ^6^	0.037	0.48 (0.28; 0.65) ^6^	<0.001
	Updating	609.00 (411.00; 664.00)	696.50 (607.25; 736.75)	463.80 (457.90; 582.50)	0.82 (0.71; 0.87) ^6^	<0.001 ^4^	0.14 (0.01; 0.37) ^6^	0.288	0.51 (0.33; 0.69) ^6^	<0.001
	Inhibition	800.00 (220.50; 800.00)	800.00 (632.25; 800.00)	514.13 (513.07; 800.00)	0.27 (0.02; 0.55) ^6^	0.089 ^4^	0.09 (0.01; 0.32) ^6^	0.522	0.28 (0.08; 0.49) ^6^	0.023
CA—Reasoning	Planning	654.50 (524.25; 694.75)	663.50 (612.00; 737.75)	513.13 (511.57; 628.00)	0.30 (0.02; 0.58) ^6^	0.099 ^4^	0.30 (0.08; 0.50) ^6^	0.022	0.45 (0.27; 0.63) ^6^	<0.001
	Processing Speed	615.50 (515.50; 700.00)	675.50 (576.25; 710.75)	597.13 (585.50; 673.00)	0.52 (0.23; 0.74) ^6^	0.003 ^4^	0.01 (0.00; 0.26) ^6^	0.917	0.20 (0.02; 0.42) ^6^	0.108
	Shifting	769.50 (730.25; 788.50)	780.00 (745.00; 796.25)	754.00 (751.20; 794.00)	0.27 (0.02; 0.56) ^6^	0.138 ^4^	0.05 (0.00; 0.28) ^6^	0.677	0.07 (0.00; 0.30) ^6^	0.634
CA—Memory	Phonological Short-Term Memory	410.00 (270.75; 592.50)	511.50 (339.25; 648.50)	362.53 (324.50; 448.00)	0.47 (0.17; 0.71) ^6^	0.007 ^4^	0.10 (0.01; 0.32) ^6^	0.468	0.27 (0.06; 0.49) ^6^	0.028
	Visual Short-Term Memory	660.00 (509.00; 703.50)	655.50 (588.25; 715.25)	511.00 (508.47; 635.50)	0.06 (0.01; 0.39) ^6^	0.747 ^4^	0.26 (0.04; 0.47) ^6^	0.037	0.31 (0.09; 0.52) ^6^	0.011
	Naming	619.50 (472.25; 677.00)	669.00 (599.75; 722.25)	496.00 (485.27; 654.00)	0.45 (0.15; 0.70) ^6^	0.009 ^4^	0.14 (0.01; 0.37) ^6^	0.284	0.32 (0.10; 0.51) ^6^	0.010
	Contextual Memory	616.00 (518.50; 675.00)	707.50 (654.25; 736.25)	489.27 (476.13; 599.50)	0.78 (0.63; 0.86) ^6^	<0.001 ^4^	0.26 (0.05; 0.46) ^6^	0.037	0.56 (0.39; 0.72) ^6^	<0.001
	Short-Term Memory	609.50 (420.75; 661.50)	623.00 (517.50; 682.00)	410.93 (409.97; 553.00)	0.31 (0.03; 0.61) ^6^	0.007 ^4^	0.26 (0.04; 0.47) ^6^	0.037	0.41 (0.19; 0.61) ^6^	0.001
	Non-Verbal Memory	650.50 (474.00; 693.25)	670.50 (626.50; 722.25)	522.47 (504.73; 691.00)	0.31 (0.03; 0.57) ^6^	0.083 ^4^	0.16 (0.01; 0.39) ^6^	0.228	0.38 (0.16; 0.55) ^6^	0.002
	Working Memory	614.50 (464.50; 660.75)	666.50 (635.75; 719.00)	445.47 (445.47; 613.50)	0.82 (0.72; 0.87) ^6^	<0.001 ^4^	0.21 (0.02; 0.43) ^6^	0.107	0.56 (0.39; 0.70) ^6^	<0.001
CA—Coordination	Response Time	338.94 ± 164.63	395.72 ± 148.40	349.32 ± 124.31	−0.35 (−0.58; −0.13) ^5^	0.005 ^3^	−0.07 (−0.51; 0.37) ^5^	0.790 ^2^	0.34 (−0.11; 0.79) ^5^	0.184 ^1^
	Hand–Eye Coordination	229.50 (125.50; 498.25)	470.50 (215.00; 619.25)	198.07 (159.50; 241.50)	0.61 (0.38; 0.77) ^6^	0.001 ^4^	0.12 (0.01; 0.36) ^6^	0.395	0.45 (0.22; 0.64) ^6^	<0.001

Data presented as M ± SD or Me (Q1; Q3), M ± SD was used when distribution in each group was normal and the difference between AN1 and AN2 had normal distribution, Me (Q1; Q3) was used otherwise. M—mean, SD—standard deviation, Me—median, Q1—first quartile, Q3—third quartile, CI—confidence interval. Comparisons were made with Student’s *t*-test for independent samples ^1^, Welch *t*-test for independent samples ^2^, Mann–Whitney U test, paired *t*-test ^3^, or Wilcoxon test ^4^. *p* values were corrected with Benjamini and Hochberg adjustment in each comparison type; this Table presents corrected *p* values (adj *p*). In the case of *t*-tests, Cohen’s D ^5^ was presented as the effect size; in case of Mann–Whitney U test/Wilcoxon, the r effect size for Wilcoxon test ^6^ was given. * Variants for paired tests. ** Variants for independent tests.

**Table 4:** Biochemical parameters in patients with low body weight (AN1), after nutritional rehabilitation (AN2), and control group (CG).

Variable	Group M ± SD/Me (Q1; Q3)			AN1 vs. AN2		AN1 vs. CG		AN2 vs. CG	
	AN1 (n = 36)	AN2 (n = 36)	CG (n = 48)	Cohen’s D/Wilcoxon Effect Size r (95% CI) *	Adj *p*	Cohen’s D/Wilcoxon Effect Size r (95% CI) **	Adj *p*	Cohen’s D/Wilcoxon Effect Size r (95% CI) **	Adj *p*
1.25(0H)2D [ng/mL] (30.0–50.0)	24.85 (17.98; 35.00)	19.65 (11.05; 26.20)	19.30 (15.18; 25.77)	0.53 (0.27; 0.75) ^6^	0.003 ^4^	0.22 (0.03; 0.43) ^6^	0.066	0.04 (0.00; 0.27) ^6^	0.835
WBC [10^3^/μL] (4.0–10.0)	4.35 (3.92; 5.03)	5.76 (5.06; 6.37)	6.65 (5.28; 7.46)	0.81 (0.68; 0.86) ^6^	<0.001 ^4^	0.60 (0.44; 0.72) ^6^	<0.001	0.26 (0.05; 0.45) ^6^	0.030
RBC [10^6^/μL] (4.0–5.0)	4.39 ± 0.56	4.33 ± 0.40	4.66 ± 0.31	0.12 (−0.22; 0.46) ^5^	0.523 ^3^	−0.63 (−1.07; −0.18) ^5^	0.030 ^2^	−0.95 (−1.40; −0.49) ^5^	<0.001 ^1^
HGB [g/dL] (12.0–16.0)	13.45 (12.90; 14.30)	12.95 (12.28; 13.55)	13.65 (13.17; 14.03)	0.28 (0.02; 0.57) ^6^	0.108 ^4^	0.06 (0.00; 0.29) ^6^	0.631	0.36 (0.17; 0.54) ^6^	0.002
HCT [%]	39.30 (37.27; 41.10)	38.65 (36.72; 39.95)	39.55 (38.65; 40.85)	0.05 (0.01; 0.40) ^6^	0.747 ^4^	0.06 (0.00; 0.30) ^6^	0.631	0.23 (0.03; 0.44) ^6^	0.054
PLT [10^3^/μL] (150–400)	226.94 ± 60.63	286.03 ± 51.03	290.35 ± 61.77	−1.03 (−1.55; −0.51) ^5^	<0.001 ^3^	−1.03 (−1.49; −0.57) ^5^	<0.001 ^1^	−0.08 (−0.51; 0.36) ^5^	0.835 ^1^
Glucose [mg/dL] (60–101)	73.17 ± 9.65	82.11 ± 5.87	84.81 ± 7.48	−1.08 (−1.61; −0.56) ^5^	<0.001 ^3^	−1.37 (−1.85; −0.89) ^5^	<0.001 ^1^	−0.40 (−0.83; 0.04) ^5^	0.108 ^1^
Insulin [mU/mL] (<15)	4.30 (2.80; 5.90)	6.15 (4.88; 9.48)	7.05 (5.30; 9.30)	0.61 (0.34; 0.83) ^6^	0.001 ^4^	0.53 (0.36; 0.67) ^6^	<0.001	0.13 (0.01; 0.33) ^6^	0.316
HOMA-IR	0.77 (0.54; 1.08)	1.23 (0.93; 2.05)	1.48 (1.13; 1.90)	0.67 (0.46; 0.83) ^6^	<0.001 ^4^	0.60 (0.46; 0.74) ^6^	<0.001	0.15 (0.01; 0.35) ^6^	0.237
TSH [mIU/L] (0.47–3.41)	2.02 ± 0.91	2.18 ± 0.99	2.14 ± 0.97	−0.17 (−0.51; 0.18) ^5^	0.375 ^3^	−0.13 (−0.57; 0.30) ^5^	0.626 ^1^	0.04 (−0.40; 0.47) ^5^	0.945 ^1^
FT4 [ng/dL] (0.86–1.37)	0.82 (0.77; 0.91)	0.79 (0.73; 0.85)	0.98 (0.93; 1.02)	0.43 (0.13; 0.68) ^6^	0.013 ^4^	0.57 (0.39; 0.73) ^6^	<0.001	0.75 (0.61; 0.83) ^6^	<0.001

Data presented as M ± SD or Me (Q1; Q3), M ± SD was used when distribution in each group was normal and the difference between AN1, and AN2 had normal distribution, Me (Q1; Q3) was used otherwise. M—mean, SD—standard deviation, Me—median, Q1—first quartile, Q3—third quartile, CI—confidence interval. Comparisons were made with Student’s *t*-test for independent samples ^1^, Welch *t*-test for independent samples ^2^, Mann–Whitney U test, paired *t*-test ^3^, or Wilcoxon test ^4^. *p* values were corrected with Benjamini and Hochberg adjustment in each comparison type; this Table presents corrected *p* values (adj *p*). In the case of *t*-tests, Cohen’s D ^5^ was presented as the effect size; in the case of Mann–Whitney U test/Wilcoxon, the r effect size for Wilcoxon test ^6^ was given. * Variants for paired tests. ** Variants for independent tests.

**Table 5:** Correlation between CAB/CA parameters and selected variables for patients with low body weight (AN1), patients after nutritional rehabilitation (AN2), and control group (CG).

Variable	CAB		CA—Perception		CA—Attention		CA—Reasoning		CA—Memory		CA—Coordination	
	rho	*p*	rho	*p*	rho	*p*	rho	*p*	rho	*p*	rho	*p*
AN1												
BDI	−0.18	0.286	−0.05	0.777	−0.18	0.290	−0.15	0.372	−0.15	0.382	−0.11	0.538
EAT-26	−0.43	0.010	−0.27	0.108	−0.29	0.090	−0.21	0.224	−0.34	0.040	−0.30	0.071
AN2												
BDI	−0.14	0.414	−0.12	0.472	−0.08	0.652	−0.19	0.266	−0.09	0.593	−0.02	0.906
EAT-26	−0.31	0.063	−0.20	0.241	−0.18	0.296	−0.07	0.666	−0.26	0.133	−0.28	0.092
CG												
BDI	−0.08	0.600	−0.10	0.532	−0.11	0.486	−0.02	0.914	−0.11	0.502	0.17	0.273
EAT-26	−0.16	0.299	−0.12	0.436	−0.35	0.024	−0.11	0.476	−0.25	0.110	0.11	0.505

rho—Spearman correlation coefficient.

## Data Availability

The datasets used and analyzed during the current study are available from the corresponding author upon reasonable request.

## Supplementary Materials

The following supporting information can be downloaded at: https://www.mdpi.com/article/10.3390/nu16203435/s1, Table S1: Linear regression outcomes for CAB in low body weight (AN1), after nutritional rehabilitation (AN2) and control group (CG).

## Abbreviations

AN	anorexia nervosa
AN1	patients in the acute phase of anorexia nervosa
AN2	patients after partial normalization of body weight on the day of discharge
CA	cognitive abilities
CAB	Cognifit Assessment Battery
ED	eating disorders
FT4	thyroxine
HCT	hematocrit
HGB	hemoglobin concentration
NR	nutritional rehabilitation
PLT	platelet count
TSH	thyrotropic hormone
WBC	white blood cell
%IBW	percentage of ideal body weight

## References

[1] Wood S., Marchant A., Allsopp M., Wilkinson K., Bethel J., Jones H., John A. (2019). Epidemiology of eating disorders in primary care in children and young people: A Clinical Practice Research Datalink study in England. BMJ Open.

[2] Petkova H., Simic M., Nicholls D., Ford T., Prina A.M., Stuart R., Livingstone N., Kelly G., MacDonald G., Eisler I. (2019). Incidence of anorexia nervosa in young people in the UK and Ireland: A national surveillance study. BMJ Open.

[3] Reas D.L., Rø Ø. (2018). Time trends in healthcare-detected incidence of anorexia nervosa and bulimia nervosa in the Norwegian National Patient Register (2010–2016). Int. J. Eat. Disord..

[4] Agostino H., Burstein B., Moubayed D., Taddeo D., Grady R., Vyver E., Dimitropoulos G., Dominic A., Coelho J.S. (2021). Trends in the Incidence of New-Onset Anorexia Nervosa and Atypical Anorexia Nervosa Among Youth During the COVID-19 Pandemic in Canada. JAMA Netw. Open.

[5] Devoe J.D., Han A., Anderson A., Katzman D.K., Patten S.B., Soumbasis A., Flanagan J., Paslakis G., Vyver E., Marcoux G. (2023). The impact of the COVID-19 pandemic on eating disorders: A systematic review. Int. J. Eat. Disord..

[6] Holland J., Hall N., Yeates D.G.R., Goldacre M. (2016). Trends in hospital admission rates for anorexia nervosa in Oxford (1968–2011) and England (1990–2011): Database studies. J. R. Soc. Med..

[7] Favaro A., Caregaro L., Tenconi E., Bosello R., Santonastaso P. (2009). Time trends in age at onset of anorexia nervosa and bulimia nervosa. J. Clin. Psychiatry.

[8] GBE—Gesundheitsberichterstattung des Bundes. https://www.gbe-bund.de/gbe/.

[9] Cruz A.M., Gonçalves-Pinho M., Santos J.V., Coutinho F., Brandão I., Freitas A. (2018). Eating disorders-Related hospitalizations in Portugal: A nationwide study from 2000 to 2014. Int. J. Eat. Disord..

[10] Fardouly J., Vartanian L.R. (2016). Social Media and Body Image Concerns: Current Research and Future Directions. Curr. Opin. Psychol..

[11] Arseniev-Koehler A., Lee H., McCormick T., Moreno M.A. (2016). #Proana: Pro-Eating Disorder Socialization on Twitter. J. Adolesc. Health.

[12] Bills E., Greene D., Stackpole R., Egan S.J. (2023). Perfectionism and eating disorders in children and adolescents: A systematic review and meta-analysis. Appetite.

[13] Vostanis P. (2024). Mental health provision for children affected by war and armed conflicts. Eur. Child Adolesc. Psychiatry.

[14] Gorrell S., Reilly E.E., Brosof L., Grange D. (2022). Le Use of Telehealth in the Management of Adolescent Eating Disorders: Patient Perspectives and Future Directions Suggested from the COVID-19 Pandemic. Adolesc. Health. Med. Ther..

[15] Muth L., Leven K.H., Moll G., Kratz O., Horndasch S. (2022). Effects of the COVID-19 Restrictions on Eating Behaviour and Eating Disorder Symptomology in Female Adolescents. Int. J. Environ. Res. Public Health.

[16] Wang S.B., Gray E.K., Coniglio K.A., Murray H.B., Stone M., Becker K.R., Thomas J.J., Eddy K.T. (2021). Cognitive rigidity and heightened attention to detail occur transdiagnostically in adolescents with eating disorders. Eat. Disord..

[17] Di Lodovico L., Versini A., Lachatre M., Marcheselli J., Ramoz N., Gorwood P. (2022). Is decision-making impairment an endophenotype of anorexia nervosa?. Eur. Psychiatry.

[18] Smith K.E., Mason T.B., Johnson J.S., Lavender J.M., Wonderlich S.A. (2018). A systematic review of reviews of neurocognitive functioning in eating disorders: The state-of-the-literature and future directions. Int. J. Eat. Disord..

[19] Lang K., Roberts M., Harrison A., Lopez C., Goddard E., Khondoker M., Treasure J., Tchanturia K. (2016). Central Coherence in Eating Disorders: A Synthesis of Studies Using the Rey Osterrieth Complex Figure Test. PLoS ONE.

[20] Fuglset T.S. (2021). Is set-shifting and central coherence in anorexia nervosa influenced by body mass index, anxiety or depression? A systematic review. BMC Psychiatry.

[21] Shott M.E., Filoteo J.V., Bhatnagar K.A.C., Peak N.J., Hagman J.O., Rockwell R., Kaye W.H., Frank G.K.W. (2012). Cognitive Set-Shifting in Anorexia Nervosa. Eur. Eat. Disord. Rev..

[22] Weider S., Indredavik M.S., Lydersen S., Hestad K. (2015). Neuropsychological function in patients with anorexia nervosa or bulimia nervosa. Int. J. Eat. Disord..

[23] Zacková M.L., Jáni M.M., Brázdil M., Nikolova Y.S., Marečková K. (2021). Cognitive impairment and depression: Meta-analysis of structural magnetic resonance imaging studies. NeuroImage Clin..

[24] American Psychiatric Association (2013). Diagnostic and Statistical Manual of Mental Disorders.

[25] Hughes E.K. (2012). Comorbid depression and anxiety in childhood and adolescent anorexia nervosa: Prevalence and implications for outcome. Clin. Psychol..

[26] Kahn M., Brunstein-Klomek A., Hadas A., Snir A., Fennig S. (2020). Early changes in depression predict outcomes of inpatient adolescent anorexia nervosa. Eat. Weight Disord..

[27] Samson K.L.I., Fischer J.A.J., Roche M.L. (2022). Iron Status, Anemia, and Iron Interventions and Their Associations with Cognitive and Academic Performance in Adolescents: A Systematic Review. Nutrients.

[28] Przybylak M., Grabowski J., Bidzan L. (2021). Cognitive functions and thyroid hormones secretion disorders. Psychiatr. Pol..

[29] Golden N.H., Cheng J., Kapphahn C.J., Buckelew S.M., Machen V.I., Kreiter A., Accurso E.C., Adams S.H., Le Grange D., Moscicki A.-B. (2021). Higher-Calorie Refeeding in Anorexia Nervosa: 1-Year Outcomes from a Randomized Controlled Trial. Pediatrics.

[30] Weir C.B., Jan A. (2024). BMI Classification Percentile and Cut Off Points. StatPearls [Internet].

[31] Nahler G. (2009). Lorentz-formula. Dictionary of Pharmaceutical Medicine.

[32] Researchers Assessment Batteries Reliability and Validity Description of CogniFit Assessment Batteries. CogniFit Assessment Battery CogniFit © 2022. https://static.cognifit.com/customersupport/Validation+for+Researchers.pdf.

[33] Rogoza R., Brytek-Matera A., Garner D.M. (2016). Analysis of the EAT-26 in a non-clinical sample. Arch. Psychiatry Psychother..

[34] (PDF) Charakterystyka Psychometryczna Polskiej Adaptacji Kwestionariusza Depresji BDI-II Aarona T. Becka (Psychometric Properties of the Polish Version of the Aaron T. Beck’s Depression Inventory BDI-II). https://www.researchgate.net/publication/269107443_Charakterystyka_psychometryczna_polskiej_adaptacji_Kwestionariusza_Depresji_BDI-II_Aarona_T_Becka_Psychometric_Properties_of_the_Polish_Version_of_the_Aaron_T_Beck’s_Depression_Inventory_BDI-II.

[35] Yanover T., Thompson J.K. (2008). Eating problems, body image disturbances, and academic achievement: Preliminary evaluation of the eating and body image disturbances academic interference scale. Int. J. Eat. Disord..

[36] Weider S., Indredavik M.S., Lydersen S., Hestad K. (2014). Intellectual function in patients with anorexia nervosa and bulimia nervosa. Eur. Eat. Disord. Rev..

[37] Sundquist J., Ohlsson H., Winkleby M.A., Sundquist K., Crump C. (2016). School Achievement and Risk of Eating Disorders in a Swedish National Cohort. J. Am. Acad. Child Adolesc. Psychiatry.

[38] Ahrén J.C., Chiesa F., Koupil I., Magnusson C., Dalman C., Goodman A. (2013). We are family—Parents, siblings, and eating disorders in a prospective total-population study of 250,000 Swedish males and females. Int. J. Eat. Disord..

[39] Ahrén-Moonga J., Silverwood R., Klinteberg B.A., Koupil I. (2009). Association of higher parental and grandparental education and higher school grades with risk of hospitalization for eating disorders in females. Am. J. Epidemiol..

[40] Lopez C., Stahl D., Tchanturia K. (2010). Estimated intelligence quotient in anorexia nervosa: A systematic review and meta-analysis of the literature. Ann. Gen. Psychiatry.

[41] Forbush K., Heatherton T.F., Keel P.K. (2007). Relationships between perfectionism and specific disordered eating behaviors. Int. J. Eat. Disord..

[42] Nikendei C., Funiok C., Pfüller U., Zastrow A., Aschenbrenner S., Weisbrod M., Herzog W., Friederich H.C. (2011). Memory performance in acute and weight-restored anorexia nervosa patients. Psychol. Med..

[43] Terhoeven V., Kallen U., Ingenerf K., Aschenbrenner S., Weisbrod M., Herzog W., Brockmeyer T., Friederich H.C., Nikendei C. (2017). Meaningful Memory in Acute Anorexia Nervosa Patients-Comparing Recall, Learning, and Recognition of Semantically Related and Semantically Unrelated Word Stimuli. Eur. Eat. Disord. Rev..

[44] Brown M., Robinson L., Campione G.C., Wuensch K., Hildebrandt T., Micali N. (2017). Intolerance of Uncertainty in Eating Disorders: A Systematic Review and Meta-Analysis. Eur. Eat. Disord. Rev..

[45] Bartholdy S., Rennalls S.J., Jacques C., Danby H., Campbell I.C., Schmidt U., O’Daly O.G. (2017). Proactive and reactive inhibitory control in eating disorders. Psychiatry Res..

[46] Fosco W.D., Hawk L.W., Colder C.R., Meisel S.N., Lengua L.J. (2019). The development of inhibitory control in adolescence and prospective relations with delinquency. J. Adolesc..

[47] Shen Y., Zhao H., Zhu J., He Y., Zhang X., Liu S., Chen J. (2020). Comparison of Intentional Inhibition and Reactive Inhibition in Adolescents and Adults: An ERP Study. Dev. Neuropsychol..

[48] Suttkus S., Schumann A., De la Cruz F., Bär K.J. (2021). Attenuated neuronal and autonomic responses during error processing in anorexia nervosa. Brain Behav..

[49] Tenconi E., Collantoni E., Meregalli V., Bonello E., Zanetti T., Veronese A., Meneguzzo P., Favaro A. (2021). Clinical and Cognitive Functioning Changes After Partial Hospitalization in Patients with Anorexia Nervosa. Front. Psychiatry.

[50] Guardia D., Carey A., Cottencin O., Thomas P., Luyat M. (2013). Disruption of Spatial Task Performance in Anorexia Nervosa. PLoS ONE.

[51] Federica S., Ilaria B., Valentina V., Leonardo M., Gianluca C., Alessandro M., Anna S. (2022). Self-perception in anorexia nervosa: When the body becomes an object. Neuropsychologia.

[52] Dann K.M., Hay P., Touyz S. (2021). Are poor set-shifting and central coherence associated with everyday function in anorexia nervosa? A systematic review. J. Eat. Disord..

[53] Lang K., Stahl D., Espie J., Treasure J., Tchanturia K. (2014). Set shifting in children and adolescents with anorexia nervosa: An exploratory systematic review and meta-analysis. Int. J. Eat. Disord..

[54] Wu M., Brockmeyer T., Hartmann M., Skunde M., Herzog W., Friederich H.C. (2014). Set-shifting ability across the spectrum of eating disorders and in overweight and obesity: A systematic review and meta-analysis. Psychol. Med..

[55] Hirst R.B., Beard C.L., Colby K.A., Quittner Z., Mills B.M., Lavender J.M. (2017). Anorexia nervosa and bulimia nervosa: A meta-analysis of executive functioning. Neurosci. Biobehav. Rev..

[56] Brooks S.J. (2016). A debate on working memory and cognitive control: Can we learn about the treatment of substance use disorders from the neural correlates of anorexia nervosa?. BMC Psychiatry.

[57] Brooks S.J., O’Daly O.G., Uher R., Schiöth H.B., Treasure J., Campbell I.C. (2012). Subliminal food images compromise superior working memory performance in women with restricting anorexia nervosa. Conscious. Cogn..

[58] Israel M., Klein M., Pruessner J., Thaler L., Spilka M., Efanov S., Ouellette A.S., Berlim M., Ali N., Beaudry T. (2015). *n*-back task performance and corresponding brain-activation patterns in women with restrictive and bulimic eating-disorder variants: Preliminary findings. Psychiatry Res..

[59] Seed J.A., McCue P.M., Wesnes K.A., Dahabra S., Young A.H. (2002). Basal activity of the HPA axis and cognitive function in anorexia nervosa. Int. J. Neuropsychopharmacol..

[60] Lao-Kaim N.P., Giampietro V.P., Williams S.C.R., Simmons A., Tchanturia K. (2014). Functional MRI investigation of verbal working memory in adults with anorexia nervosa. Eur. Psychiatry.

[61] De Filippo E., Marra M., Alfinito F., Di Guglielmo M.L., Majorano P., Cerciello G., De Caprio C., Contaldo F., Pasanisi F. (2016). Hematological complications in anorexia nervosa. Eur. J. Clin. Nutr..

[62] Mamou G., Sider A., Bouscary D., Moro M.R., Blanchet-Collet C. (2016). Anemia in Anorexia Nervosa: The Best Way to Deal with it—An Overview of Literature. J. Hum. Nutr. Food Sci..

[63] Hütter G., Ganepola S., Hofmann W.K. (2009). The hematology of anorexia nervosa. Int. J. Eat. Disord..

[64] Mehler P.S., Blalock D.V., Walden K., Kaur S., McBride J., Walsh K., Watts J. (2018). Medical findings in 1026 consecutive adult inpatient-residential eating disordered patients. Int. J. Eat. Disord..

[65] Gibson D., Gibson D., Watters A., Watters A., Cost J., Cost J., Mascolo M., Mehler P.S., Mehler P.S., Mehler P.S. (2020). Extreme anorexia nervosa: Medical findings, outcomes, and inferences from a retrospective cohort. J. Eat. Disord..

[66] Matsunaga H., Riku K., Shimizu K., Fujimi S. (2024). Severe hypoglycemia with reduced liver volume as an indicator of end-stage malnutrition in patients with anorexia nervosa: A retrospective observational study. J. Eat. Disord..

[67] Misra M., Klibanski A. (2010). Neuroendocrine Consequences of Anorexia Nervosa in Adolescents. Endocr. Dev..

[68] DiVasta A.D., Feldman H.A., Brown J.N., Giancaterino C., Holick M.F., Gordon C.M. (2011). Bioavailability of vitamin D in malnourished adolescents with anorexia nervosa. J. Clin. Endocrinol. Metab..

[69] Tasegian A., Curcio F., Dalla Ragione L., Rossetti F., Cataldi S., Codini M., Ambesi-Impiombato F.S., Beccari T., Albi E. (2016). Hypovitaminosis D3, Leukopenia, and Human Serotonin Transporter Polymorphism in Anorexia Nervosa and Bulimia Nervosa. Mediators Inflamm..

[70] Libuda L., Timmesfeld N., Antel J., Hirtz R., Bauer J., Führer D., Zwanziger D., Öztürk D., Langenbach G., Hahn D. (2020). Effect of vitamin D deficiency on depressive symptoms in child and adolescent psychiatric patients: Results of a randomized controlled trial. Eur. J. Nutr..

[71] Föcker M., Timmesfeld N., Bühlmeier J., Zwanziger D., Führer D., Grasemann C., Ehrlich S., Egberts K., Fleischhaker C., Wewetzer C. (2021). Vitamin d level trajectories of adolescent patients with anorexia nervosa at inpatient admission, during treatment, and at one year follow up: Association with depressive symptoms. Nutrients.

[72] Hemmingsen S.D., Lichtenstein M.B., Hussain A.A., Sjögren J.M., Støving R.K. (2020). Case report: Cognitive performance in an extreme case of anorexia nervosa with a body mass index of 7.7. BMC Psychiatry.

[73] Laczkovics C., Czernin K., Carlitscheck J., Zeiler M., Schlund P., Wunram H.L., Lehmkuhl G., Krischer M. (2023). Personality Disorder in Adolescent Patients with Anorexia Nervosa. Psychopathology.

