# Supplementary material for: Cognitive Functions in Adolescent Girls with Anorexia Nervosa during Nutritional Rehabilitation
Source: Nutrients. 2024 Oct 10;16(20):3435. doi: 10.3390/nu16203435 (PMC11510226; doi:10.3390/nu16203435)
Supplement: Supplementary file 1 [file nutrients-16-03435-s001.zip › nutrients-3192481-supplementary.pdf]

Table S1. Linear regression outcomes for CAB in low body weight (AN1), after nutritional rehabilitation (AN2) and control group (CG)

Variable	AN1 (n = 36)				AN2 (n = 36)				CG (n = 48)			
	β	95% CI dla β	Std. β	p	β	95% CI dla β	Std. β	p	B	95% CI dla β	Std. β	p
Univariate regression models												
Age	-10.88	-31.00 to 9.24	-0.19	0.280	-10.20	-25.36 to 4.95	-0.23	0.180	-16.54	-41.73 to 8.65	-0.20	0.192
Height [m]	-305.25	-613.59 to 3.09	-0.33	0.052	-215.48	-451.83 to 20.86	-0.30	0.073	129.73	-439.99 to 699.45	0.07	0.648
BMI [kg/m²]	-21.47	-43.58 to 0.65	-0.32	0.057	-0.88	-17.31 to 15.55	-0.02	0.914	5.47	-5.29 to 16.24	0.16	0.311
Body weight [kg]	-7.80	-13.07 to -2.54	-0.46	0.005	-2.49	-5.91 to 0.94	-0.25	0.150	2.70	-1.36 to 6.77	0.21	0.187
IBW	-6.11	-12.27 to 0.06	-0.33	0.052	-4.31	-9.04 to 0.42	-0.30	0.073	2.59	-8.80 to 13.99	0.07	0.648
%IBW	-541.94	-974.81 to -109.07	-0.40	0.016	-104.67	-411.69 to 202.35	-0.12	0.493	133.86	-101.95 to 369.66	0.18	0.258
1,25(0H)2D [ng/ml] (30-50)	-0.70	-3.10 to 1.69	-0.10	0.554	0.77	-1.58 to 3.12	0.11	0.511	-0.45	-2.53 to 1.63	-0.07	0.662
WBC [10^3/ul] (4-10)	-14.59	-47.18 to 17.99	-0.15	0.369	12.21	-3.00 to 27.43	0.27	0.112	2.35	-10.33 to 15.04	0.06	0.710
RBC [10^6/ul] (4-5)	-20.74	-78.88 to 37.40	-0.12	0.473	28.42	-33.20 to 90.03	0.16	0.355	19.06	-59.11 to 97.23	0.08	0.625
HGB [g/dl] (12-16)	-7.63	-26.94 to 11.67	-0.14	0.427	6.33	-10.25 to 22.91	0.13	0.443	0.56	-31.08 to 32.21	0.01	0.971
HCT [%]	-2.05	-9.51 to 5.41	-0.10	0.580	0.59	-0.76 to 1.93	0.15	0.382	-1.90	-13.52 to 9.73	-0.05	0.743
PLT [10^3/ul] (150-400)	-0.05	-0.59 to 0.48	-0.03	0.841	-0.20	-0.68 to 0.28	-0.15	0.397	0.03	-0.38 to 0.43	0.02	0.892
Glucose [mg/dl] (60-101)	1.15	-2.20 to 4.51	0.12	0.490	-2.83	-6.94 to 1.29	-0.23	0.172	2.77	-0.41 to 5.96	0.26	0.086
Insulin [mU/ml] (<15)	0.71	-9.66 to 11.09	0.02	0.890	-5.03	-12.21 to 2.15	-0.24	0.164	-1.29	-7.88 to 5.30	-0.06	0.695

HOMA-IR	5.60	-60.19 to 71.38	0.03	0.864	-26.94	-60.97 to 7.09	-0.27	0.117	-1.29	-30.82 to 28.24	-0.01	0.930
FT4 [ng/dL] (0,86-1,37)	3.35	-288.61 to 295.31	0.00	0.982	-10.49	-252.20 to 231.22	-0.02	0.930	195.80	-136.67 to 528.27	0.18	0.241
TSH [mIU/l] (0,47-3,41)	2.25	-33.55 to 38.05	0.02	0.899	-0.23	-25.30 to 24.83	0.00	0.985	28.22	4.46 to 51.97	0.35	0.021
BDI	-1.37	-3.93 to 1.19	-0.18	0.285	-0.83	-2.68 to 1.03	-0.15	0.370	-0.48	-2.90 to 1.94	-0.06	0.692
EAT-26	-2.77	-4.85 to -0.69	-0.42	0.011	-1.38	-2.86 to 0.10	-0.31	0.066	-1.14	-3.44 to 0.63	-0.22	0.170
Multivariate regression models – version 1*												
Age [years]	-8.19	-26.67 to 10.29	-0.14	0.374	-11.69	-27.55 to 4.17	-0.26	0.143	-13.36	-42.03 to 15.30	-0.16	0.351
BMI [kg/m²]	-14.60	-36.32 to 7.12	-0.22	0.180	0.13	-16.99 to 17.24	0.00	0.988	3.01	-9.11 to 15.12	0.09	0.618
EAT-26	-2.34	-4.47 to -0.21	-0.36	0.032	-1.49	-3.00 to 0.02	-0.33	0.053	-1.41	-3.45 to 0.63	-0.22	0.170
Multivariate regression models – version 2**												
Body weight [kg]	-5.83	-11.63 to -0.03	-0.34	0.049	-2.85	-6.14 to 0.44	-0.28	0.087	2.26	-1.79 to 6.31	0.19	0.267
Glucose [mg/dl] (60-101)	0.51	-2.53 to 3.55	0.05	0.733	-2.12	-6.20 to 1.97	-0.17	0.299	2.40	-0.83 to 5.63	0.22	0.141
EAT-26	-1.72	-4.00 to 0.56	-0.26	0.135	-1.27	-2.77 to 0.24	-0.28	0.096	-1.16	-3.17 to 0.86	-0.18	0.252

β – linear regression coefficient, Std. β – standardized linear regression coefficient, CI – confidence interval.

* Multivariate models version 1 assumed the initial independent variables: age, BMI, %IBW, glucose, HOMA, EAT-26, and BDI.

* Multivariate models version 2 assumed the following initial set of the independent variables: age, body weight, %IBW, glucose, HOMA, EAT-26, BDI.
